# Measuring therapeutic engagement in acute mental health inpatient environments: the perspectives of service users and mental health nurses

**DOI:** 10.1186/s12888-021-03561-z

**Published:** 2021-11-08

**Authors:** M. Chambers, S. McAndrew, F. Nolan, B. Thomas, P. Watts, X. Kantaris

**Affiliations:** 1 Kingston University and St George’s, University of London, Faculty of Health, Social Care and Education, St George’s Campus, 6th Floor Hunter Wing, Cranmer Terrace, London, SW17 0RE UK; 2 University of Salford, School of Health and Society, Salford, Greater Manchester, M6 6PU UK; 3 Anglia Ruskin University, Faculty of Health, Education, Medicine and Social Care, Bishop Hall Lane, Chelmsford, Essex CM1 1SQ UK; 4 London Southbank University, 103 Borough Road, London, SE1 0AA UK; 5 Formerly of Somerset Partnership NHS Foundation Trust, Community Mental Health Nursing, 2nd Floor Mallard Court, Express Park, Bristol Road, Bristol, TA4 4RN UK

**Keywords:** Acute, Inpatient, Registered mental health nurses, Service users, Therapeutic engagement

## Abstract

**Background:**

A key component of caring for service users (SUs) in acute mental health inpatient environments is Therapeutic Engagement (TE). To that end, the Therapeutic Engagement Questionnaire (TEQ) was developed and validated. The TEQ measures TE between SUs and registered mental health nurses (RMHNs) from the perspective of both parties and can quantify and recognise how nurses engage with SUs and monitor this activity as well as its enhancement of SU care and recovery. The aim of this study was to explore the views of SUs and RMHNs in relation to the TEQ and how it could be adopted into clinical practice within an acute inpatient environment.

**Methods:**

As part of the validation stage of the development of the TEQ, the views of 628 SUs and 543 RMHNs were collected using a qualitative approach by way of free text at the end of the questionnaire. Two questions required free text response: – ‘what do you think of the TEQ?’, and ‘how can it be utilised?’

**Results:**

Following thematic analysis, it was found that both sets of participants stated that such a tool could be utilised to improve the service, could help nurses with reflective practice, be utilised as part of clinical supervision and to aid nurses’ professional development. The nurse participants also stated that such a tool would help track SU participation and enablement in their care. Furthermore, the nurses noted that the tool would help to reinforce the core ‘caring’ value of nursing and the overall goal of recovery. The SUs added that the TEQ would recognise the work of mental health nurses and provide them with a clear opportunity to express their views in relation to nursing staff.

**Conclusions:**

Therapeutic engagement (TE) has been identified as part of the repertoire of mental health nursing and both groups of participants identified how a tool to assess this construct may be utilised in day-to-day clinical practice to the benefit of each group.

## Background

Therapeutic engagement (TE) is at the core of quality mental health nursing and has been recognised as such since the work of Peplau (1952) [1]. Being able to engage with SUs and communicate effectively is a fundamental skill required of all nurses [2].

Therapeutic engagement (TE) can be viewed as fundamental to mental health nursing. The relevance of communication and TE in mental health care is also emphasised in the Chief Nursing Officer's review of mental health nursing [3] in which a key recommendation to improving outcomes for SUs is to develop and sustain positive TE. Therapeutic engagement (TE) is viewed as a partnership relationship between the RMHN and SU with shared decision-making, recovery focused goals based on mutual trust, respect, and negotiation, enabling SUs to problem-solve and enhance their coping capacity [4]. For clarity, ‘recovery’ in the context of this study is loosely defined to be the significant decrease/absence of clinical symptoms, decreases in duration and rate/number of hospital (re) admissions. The authors acknowledge that the concept itself is a process and can be subjective and includes agents such as hope and empowerment. There have been many interpretations and definitions of TE [5]. Whilst there appears to be no universal definition of TE, words such as ‘healing’, ‘benefit’, ‘empowerment’ and ‘restorative’ are concepts highlighted in its surrounding discussions. Therapeutic interpersonal relationships appear to be the primary component of all healthcare interactions that facilitate the development of positive ‘clinician–patient’ experiences [6]. With an increasing focus on ‘patient-centered’ care, some authors have highlighted the importance of healthcare professionals therapeutically engaging with SUs in improving health-related outcomes [7].

Engagement in treatment has been identified as key to its effectiveness [8]. Given its multifaceted nature authors have advocated that, those working in the field of mental health nursing should consider the impact of both the therapeutic environment and atmosphere and 1:1 session on the support and care available to SUs [9, 10].^.^ TE is viewed an interpersonal construct, characteristic of nurses’ approach towards SUs and known to impact on care quality and recovery [11]. The therapeutic relationship has potentially been known to be affected by administrative issues and time constraints which detract from nurses’ capacity to maintain therapeutic relationships with SUs [12]. McAllister (2017) [5] reported potential for disparity between actual and desirable levels of TE and advocated an emphasis on TE in nurse education, ward management and clinical supervision.

This study aimed to explore the views of SUs and RMHNs in relation to the TEQ and how it could be adopted into clinical practice within an acute inpatient environment. It was the intention of the authors for the study to be specifically about the TEQ as a tool and not about TE as a phenomenon or TE tools in general.

## Methods

### Study context

This questionnaire evaluation of user experiences and opinions study formed part of the development and validation of the Therapeutic Engagement Questionnaire (TEQ), a TE metric developed in partnership with SUs and RMHNs [13, 14]. The TEQ was developed in accordance with psychometric theory. The data collected underwent a Principal Components Analysis (PCA), adequate for the development of a measurement tool, and most used in exploratory factor analysis to determine underlying domains (factors and structural validity) of measurement tools. The TEQ has two versions; one for SUs and one for RMHNs, each scored within two contexts – 1:1 interaction between these two parties and the overall environment and atmosphere of the ward/unit. The TEQ has 20 items, scored on a four-point Likert scale (strongly disagree-strongly agree), and incorporates two sub-scales (care interactions and care delivery). The TEQ has been shown to have sound psychometric properties [14].

### Study sites

The NHS Trusts providing mental health inpatient care in England were approached to participate. General adult acute wards within the organisations were eligible to participate. A national sample of 628 SUs and 543 RMHNs was recruited across 26 England Mental Health Trusts with wide geographical spread.

### Sampling, recruitment, and participants

The details that follow applied in the first instance to initial work surrounding the development and validation of the TEQ [14]. Purposive sampling was adopted. Ward managers identified eligible SUs and RMHNs for the study who were then invited by the research team to participate. Data were collected within a 6-month period (June 2018-January 2019). Adult service users (18+) with the following eligibility criteria were invited to complete the SU version of the questionnaire within their care environment (with support from a person of their choice if needed who was not a staff member): residing for more than one week within an adult acute inpatient mental healthcare setting, mental capacity to consent (as determined by the ward nursing staff and treating Psychiatrist using the four-point British Medical Association mental capacity test) and good command of the English language as the TEQ has been initially developed in English. Registered mental health nurses working in an acute inpatient mental health setting attached to one of the 26 Mental Health NHS Trust participating in the study with a permanent work contract were invited to complete the nurse version of the questionnaire within their work environment. Table 1 shows the participant characteristics which appeared to cover the diversity of SUs and nurses. This table is also cited in a previous paper that fully describes the development and validation of the TEQ (Chambers et al 2019) in accordance with psychometric theory.

**Table 1:** Characteristics of the participants

Variable	Service users	Nurses
n	628	543
Gender^a^		
Female	50	35
Male	33	8
Not stated	575	500
Ethnicity		
White British	28	40
Black or Black mixed	8	25
Asian or Asian mixed	7	5
Not stated	585	473
Age^b^		
20–30	–	38
31–40	–	19
41–50	–	13
51–60	–	5
61–70	–	1
Not stated	–	467
Education^b^		
Higher degree	–	7
University degree	–	48
University diploma	–	4
Other	–	5
Not stated	–	479
Grade		
Band 5		38
Band 6		13
Band 7		4
Band 8		1
Not stated		487

^a^All values from here onwards are in %

^b^Not collected for SUs

### Data collection procedures

As part of the validation of the TEQ, the views of the SUs and RMHNs were collected using a qualitative approach via two free text focused questions at the end of the questionnaire which they completed within their care or work environment. These questions were - ‘*what do you think of the TEQ?*’, and *‘how can it be utilised?’*. This study was informed by a previous qualitative study [8] which explored the element of engagement by asking participants to reflect on their experiences. Free text questions are typically utilised to ask for people’s opinions, or to provide them with the opportunity to explain a previous answer. Service users (SUs) could complete the TEQ and free text boxes with support from a person (if they wanted) but not a member of the ward team. In the free text boxes, the SUs and nurses were asked to say what they thought of the TEQ and how it could be utilised in day-to-day clinical practice within an acute inpatient care environment. Participants were encouraged to express their opinions and ideas and to be as honest as possible. All participants were assured anonymity and that their answers would remain confidential. Both groups of participants gave their perspectives after the SUs were resident in the study setting post 1 week.

Nurses in management positions who worked at the host organisations helped facilitate the data collection – one per ward. They would have been aware of the delivery of care to the service users recruited to the study and were indeed colleagues to the staff recruited.

### Data analysis

The data were thematically analysed using the six-phase approach described by Braun and Clarke (2013) [15]. Thematic analysis is an effective and explicit approach when research is addressing probing questions. It is a flexible yet clearly defined approach that provides an accessible method of analysis to qualitative researchers [16]. The process involved becoming familiar with the data, generating initial codes, searching for themes, reviewing the themes, defining, and naming themes and producing the analysis. No pre-determined codes were used and, alternatively, the researchers conceptualised the themes throughout the analytic process in a data-led approach. This resulted in the conceptualisation of the themes. All data were analysed by XK and a random sample of the data (10%) reviewed by each author to ensure trustworthiness [17] and credibility [18]. After consultation, refinements to the coding were minimal and the data codes were integrated into broad themes. The inductive themes that emerged during analysis were captured using key words. Emergent themes were broad and naturally formed. The themes were clear from the content of the free text responses from both SUs and RMHNs. It should be known that all statements made by the two groups of participants were accounted for in the analysis and ambiguity in statement meaning (uncertainties related to what participants meant by their answers) was not uncovered during analysis. Both groups of participants were broad in their statements rather than specific.

## Results

In total, 628 SUs and 543 nurses completed the appropriate version of the TEQ within 26 participating Mental Health Trusts with wide geographical spread across England; that is 48% of the total number of Mental Health Trusts in England. It should be known that not all nurses and SUs responded to both free text questions. The proportions were not calculated neither were the number of eligible RMHNs working in the Trusts nor the number of eligible SUs residing on the wards. The transient nature of mental health nursing made this difficult.

Participants’ responses were rich in content and reflective in nature. Themes were identified within and across each participant group; the nurse’s data appeared to be divided into two themes *(Service user participation and enablement in care, and, Reinforcing the core value of caring and recovery),* Service Users (SUs) identified one theme, *(Recognition of work and staff appreciation),* and both groups spoke about one common theme, *(Reflective tool to aid clinical supervision, training, and service development).*

Whilst it was the intention of the authors for SUs and RMHNs to answer the study’s two focused questions the results also appear to reflect themes related to other things including what TE means to them and general reflection about TE in general. The themes that followed the data analysis are presented below.

### Nurses’ views

#### Theme: service user participation and enablement in care

Empowerment of SUs is clearly a way to ensure the service user is at the centre of their own care [19]. The nurses who participated stated this as a reason to implement a tool to assess TE, believing it to be a way of identifying how much SUs are actually (wanting) to participate in their care and how it facilitates their recovery – “*The information provided could be used to promote and enhance better standard of care for SUs. It enables SUs participation and enablement”*. Such a tool can also *“identify the difference between the standard of care nurses give to their named patients compared to patients in general”* helping to identify the influence and function of a mental health nurse. The tool can be utilised to deduce the impact of TE on the SU and the nurses – *“It may help to deduce the advantage of the engagement of staff with SUs”.*

#### Theme: reinforcing the core value of caring and recovery

Nurses identified that the TEQ can reinforce the multifaceted core ‘care’ element of nursing. Being a professional nurse means that the SUs in your care must be able to trust you, and that you treat them with dignity, kindness, respect, and compassion. It means understanding the NMC code of conduct - meaning that nurses need to be held accountable. These findings concur with work by Rowan (2010) [20]. According to the nurse participants, the TEQ can, *“make sure basic care values are in place”; “to show how nurses are feeling towards patients that are in our care. How we view their goals, wishes and dreams about their future.”*

The goal of care appeared to be at the forefront of the mind of many nurse participants. Some stated that the TEQ could, *“gauge how effectively healthcare professionals feel they impact on SU’s recovery in a positive manner”* and “*improve recovery to prevent relapse rates”*; *“I think SUs should be first and foremost be considered as a valuable person and to be given all the respect and dignity. The care we provide should be satisfactory and realistic. Staff should be honest to clients. Their views and wishes must be considered. Short term and long-term goals to be always considered.”*

### Service users’ views

#### Theme: recognition of work and staff appreciation

The SUs stated that a tool to assess TE will show the work that is undertaken by a RMHN and it would provide a ‘feedback’ opportunity to appreciate or critique the staff who have cared for them – *“To let the staff know they are appreciated and that they are doing a good job and providing much needed care in a crisis. And is a model for other units to follow. Also, if any problems are indicated there can be amended or worked on”*; *“It could be used in the recognition of the good work that the nurses and staff do”* and to “help quantify nursing input.” The TE relationship between nurses and SUs can be complex; it was mentioned that the “tool can also be utilised to “*improve the dialogue between staff and patients re their care plan and access to important information.”*

#### Shared views across both groups

#### Theme: reflective tool to aid clinical supervision, training, and service development

The need for a tool to assess TE was dominant amongst both groups of participants. They saw the tool as a way to reflect on nursing practice and to identify areas for nurse training and development, with the overall goal being to improve the service provided to SUs. In particular, there was a desire to use the tool to improve the quality of the service provided by RMHN and improve the service user experience.

The tool would be, “*Useful as a reflective exercise for nurses to think about their internal attitudes towards their service users”* (nurse) and *“Useful as an evaluation tool for how the care process between service user and named nurse is”* (SU). *“I think nurses need more protected reflective space to think about the work they do with patients”* (nurse), *“to improve services and help other patients - to continually improve services in the never-ending improvement of the NHS”* (SU). *“The information could also help to highlight the areas of improvement regarding therapeutic environment in hospital settings”* (nurse). Improvements needed may *“identity skills which are lacking or time and resource issues” (SU)* which may be addressed by senior personnel.

Overall, the TEQ was viewed as a positive tool; *“the information from the questionnaires (tool) can be used to improve therapeutic engagement. May need training and skills workshop”* (SU). *“It may highlight need for more awareness and support (of service users)”* (nurse). The tool may be utilised for *“personal development, personal reflection to compare nurses’ experiences with SU experiences in how they feel they are being treated. What the expectations of SUs are. Do all acute staff have the same values, and do they all feel they work the same? Do staff feel they are meeting SU needs?”* (SU). *“Hopefully, it can help to identify the best methods of building/maintaining a therapeutic relationship and improve engagement. Also, help to reduce time nurses spend on tasks that do not improve therapeutic engagement”* (nurse).

It should be noted that whilst both groups of participants were asked questions pertaining to the TEQ and its use, the participants also shared their views on the benefits of having TE, while the nurses articulated how certain circumstances may hinder the process of TE.

Both sets of participants stated the following benefits of TE:

- 1:1 time with SUs is very important and aids recovery - *“1:1s are very good befitting the patients’ recovery and time spent in hospital”* (nurse); *“1:1 session and my general interactions with staff override any systematic and bureaucratic operations and as such are valuable”* (SU).
- Nurses should be caring and communicative – “*Nurses should have a vested interest in caring for people such as service users” (nurse). “The nurses have shown me that they are professional with good communication skills”* (SU).
- TE should be service user-centred - *“Caring for patient need, to be patient-centred care, taking into account patient’s choice is so important; the patient should be in control. You as a named nurse should flow with them. They should lead in the planning of the care plan”* (nurse); *“Discussing care plans with service users is so important”* (SU).

Nurses stated that TE can be hindered by the challenges of time and resources - “*Don’t get time to have 1:1 time as often as I’d like due to organisation and running of the ward”*; *“Staff shortages often refrain the nurses from engaging in therapeutic time with the patients. The fact that the nurse cannot spend the time with patients greatly reduces the trust and therapeutic alliance with them.”* The amount of paperwork required - *“For me personally, there’s sometimes too much paperwork for staff and because of that there’s less time for patients”; “I believe generally speaking, myself and my colleagues currently feel unable to provide a sufficient level of therapeutic input with clients due to pressures like paperwork.”*

## Discussion

The development of the tools to measure TE is not only important for clinical practice and clinical research [21] but the development of such measures will potentially allow nurses to identify SUs who are difficult to engage with quickly and effectively [22], provide outcome measures in evaluations designed to enhance future TE, and identify factors that are associated with successful and unsuccessful engagement in treatment [23] and ultimately help nurses to do their job. Patient related characteristics, like language used and misconceptions, and nurse related characteristics, like an ‘all-knowing’ attitude, may impact TE, as well as additional environmental-related issues that may play a role in a lack of effective therapeutic communication among patients and nurses.

Ultimately, a tool to measure TE within adult acute mental health inpatient environments can identify and quantify the nature of therapeutic interaction between nurse and SU and make more explicit and visible the skills and value of RMHNs, something which Brown and Fowler (1979) [24] identified as lacking over 40 years ago.

Both groups viewed TE as a necessity within the nurse-SU dynamic in acute mental healthcare environments This study edges towards identifying how a measure that assesses TE can be utilised in day-to-day practice from the perspective of the people that the service ‘serves’ and those who work within it.

Understanding the importance TE has from the perspective of nurses and the SUs they care for, can be a powerful catalyst for change within mental health practice. It must be acknowledged that barriers to TE extend beyond time and paperwork. Providing recovery person-centred care is so important and it is vital that barriers are addressed at both the macro health services level, as well as the micro client-service user interface level.

More time with SUs will enable better TE and this is the forever perpetual issue. Commitment to partnership working is dependent on many elements coming together. The challenges nurses identified to providing TE in acute care environments must be acknowledged and addressed if the goals of care and treatment can be fulfilled within a clinical recovery framework (as loosely defined by the authors earlier). It is clear from the results of this study that whilst nurses and SUs can have differing perceptions of the purpose of TE, there was a shared consensus of what is required to build and maintain TE amongst RMHNs and SUs. If poor TE is to be effectively addressed, it is imperative that conceptually and psychometrically sound generalisable measures of this construct are utilised and recognised in day-to-day clinical practice.

The views of both sets of study participants confirm the content of the TEQ as valid, as previously reported by Chambers et al. (2020) [14]. Understanding the challenges to TE in acute mental health care settings and day to day clinical practice can lead to service changes, which would benefit both nursing staff and the people they support and care for.

### Limitations

This study included 26 NHS Mental Health Trusts in England with a wide geographical spread and the authors believe that the study can provide some insight into the elements of TE, its constraints and perceived effectiveness of using a tool to measure TE in mental health environments. However, the study findings may be specific to the nurses and SUs working and residing in/at the participating Trusts and the nurse-SU dynamic delivered in those Trusts. We do not know how representative our sample is of the overall staff or patient population as we have not gathered comparative workforce or patient throughput data. In addition, the questions were brief and did not allow for further exploration and/or probing of participants’ thoughts and responses nor were their ways to ensure what the participants understood by the questions and thus what they were answering. As the questions were part of a written survey, rather than qualitative interviews, the risk that the individual participants gave the questions different meaning depending on context and thereby answered to different things was very high. A more in-depth national TEQ implementation programme is underway that aims to provide further insight into its use.

The proportion of the SU participants who were assisted to complete the questionnaire by a researcher is not known. This bias must be recognised as this assistance may have affected their responses.

Service users may have felt compelled (not from overt pressure from the researcher, but pressure which was inherent in the situation) to provide positive replies. The responses were largely positive. This would indicate that the participants were a self-selecting group of people who had positive experiences in their work or treatment and may have been more likely to provide positive responses to questionnaires. There is very limited, if any, presentation of critical viewpoints. This could indicate that those who answered the two free text questions were a self-selecting group who may have felt more positive towards their work and treatment experiences. Furthermore, the thematic analysis is subjective, and the interpretation of the free text is the perception of the authors.

## Conclusions

The TEQ can determine the nature of therapeutic engagement between RMHNs and SUs and can provide evidence to healthcare employers in relation to the value of work undertaken by mental health nurses. This tool can clearly be utilised in day-to-day clinical practice within acute mental health care environments. The views of SUs receiving inpatient care and those of the registered nursing staff who ‘front’ these services must be considered along with other professions to ensure compassionate, and effective care, and treatment. The need and significance of TE to SUs and nurses is clear. It is evident that the TEQ as a resource can facilitate the recognition and value of this core component of mental health nursing.

## Data Availability

The datasets used and analysed for the study are not publicly available due to sensitive and personal information disclosed during the data collection. De-identified data are available from the corresponding author on reasonable request.

## Abbreviations

RMHN: Registered Mental Health Nurse
SU: Service User
TE: Therapeutic Engagement
TEQ: Therapeutic Engagement Questionnaire
